# Low Intensity Focused tDCS Over the Motor Cortex Shows Inefficacy to Improve Motor Imagery Performance

**DOI:** 10.3389/fnins.2017.00391

**Published:** 2017-07-06

**Authors:** Irma N. Angulo-Sherman, Marisol Rodríguez-Ugarte, Eduardo Iáñez, Jose M. Azorín

**Affiliations:** ^1^Monterrey's Unit, Biomedical Signal Processing Laboratory, Center for Research and Advanced StudiesApodaca, Mexico; ^2^Brain-Machine Interface Systems Lab, Systems Engineering and Automation Department, Universidad Miguel Hernández de ElcheElche, Spain

**Keywords:** BCI, tDCS, motor imagery, current density, neurorehabilitation, 4 × 1 ring

## Abstract

Transcranial direct current stimulation (tDCS) is a brain stimulation technique that can enhance motor activity by stimulating the motor path. Thus, tDCS has the potential of improving the performance of brain-computer interfaces during motor neurorehabilitation. tDCS effects depend on several aspects, including the current density, which usually varies between 0.02 and 0.08 mA/cm^2^, and the location of the stimulation electrodes. Hence, testing tDCS montages at several current levels would allow the selection of current parameters for improving stimulation outcomes and the comparison of montages. In a previous study, we found that cortico-cerebellar tDCS shows potential of enhancing right-hand motor imagery. In this paper, we aim to evaluate the effects of the focal stimulation of the motor cortex over motor imagery. In particular, the effect of supplying tDCS with a 4 × 1 ring montage, which consists in placing an anode on the motor cortex and four cathodes around it, over motor imagery was assessed with different current densities. Electroencephalographic (EEG) classification into rest or right-hand/feet motor imagery was evaluated on five healthy subjects for two stimulation schemes: applying tDCS for 10 min on the (1) right-hand or (2) feet motor cortex before EEG recording. Accuracy differences related to the tDCS intensity, as well as μ and β band power changes, were tested for each subject and tDCS modality. In addition, a simulation of the electric field induced by the montage was used to describe its effect on the brain. Results show no improvement trends on classification for the evaluated currents, which is in accordance with the observation of variable EEG band power results despite the focused stimulation. The lack of effects is probably related to the underestimation of the current intensity required to apply a particular current density for small electrodes and the relatively short inter-electrode distance. Hence, higher current intensities should be evaluated in the future for this montage.

## 1. Introduction

Transcranial direct current stimulation (tDCS) is a noninvasive technique for the temporal modulation of brain excitability with direct current (Foerster et al., 2013). This technique has shown potential for improving motor performance (Reis and Fritsch, 2011), so its implementation is promising for motor neurorehabilitation. However, the effects of tDCS depend on the intensity of the stimulation, the configuration of the electrode array, and the size of the electrodes that are used for tDCS supply, among other factors (Nitsche et al., 2008). In order to improve the focalization of the stimulation and the reproducibility of results, the use of high-definition tDCS (HD-tDCS) with smaller electrodes has gained popularity (Woods and Martin, 2016). In particular, some studies use a 4 × 1 ring montage, which consists on placing an electrode over a target brain region and four return electrodes around it (Villamar et al., 2013). With regard to the strength of tDCS, the current density is the variable that is used to infer the efficacy of stimulation, and it is estimated as the ratio of current intensity and electrode size (*I*/*A*) (Nitsche et al., 2007). Most studies use a current density within the range of 0.02 and 0.08 mA/cm^2^ (Nitsche et al., 2008), where 0.028 mA/cm^2^ is the recommended limit in terms of safety and comfort (Bikson et al., 2016). Nevertheless, some studies evaluate greater current densities according to the *I*/*A* ratio, as it can be found in Datta et al. (2012), Minhas et al. (2012), and Roy et al. (2014). In addition, it should be considered that safety parameters may be protocol-specific and that the supply of HD-tDCS with an intensity of 2 mA and a duration of 20 minutes is reported to be still tolerable (Bikson et al., 2009; Villamar et al., 2013).

Recent studies such as Sharma and Baron (2013) indicate that the performance of motor imagery and actual movement activates common neural networks. Also, it is widely known that both motor tasks are associated to the attenuation of the power of electroencephalographic signals (EEG) at μ (8–12 Hz) and β (13–30 Hz) bands (Neuper et al., 2005), which is produced by the decrease of synchronization of neuronal signals (event-related desynchronization or ERD). Hence, rehabilitation research in the field of brain-computer interfaces (BCIs) includes motor imagery as a mechanism for inducing plasticity by allowing the repetitive mental practice of motor tasks (Ang et al., 2012).

This study assesses the effect of supplying different tDCS intensities over EEG classification into either rest or feet/right-hand motor imagery for two tDCS modalities that use a 4 × 1 ring-based montage: providing anodal tDCS for 10 min over the (1) feet or (2) right-hand motor cortex before EEG recording. The current intensities evaluated in the present study approximated current density values (0.02–0.06 mA/cm^2^, according to the *I*/*A* ratio) within the range used in most tDCS studies. In this case, classification improvement after tDCS supply is studied by comparing, through statistical tests, the classification accuracy that was obtained in different sessions where a specific value of current was applied. Then, the change on μ and β band power when a particular current is supplied respect to the case of providing no stimulation was analyzed in order to obtain more information about the EEG changes that are associated to the possible classification trends. Also, a simulation of the electric field that is induced by the tDCS montage was used to describe its focused effect on the brain. In addition, we have further compared our results with our previous findings (Angulo-Sherman et al., 2017), which evaluated the EEG classification improvements when the same approximated current densities were provided but using a montage that was aimed to influence the cortico-cerebellar motor path by positioning the stimulation electrodes over the motor cortical area and the cerebellum. The present work was performed with the main goal of describing the effect of the most common current densities over the performance of a motor task (i.e., motor imagery) when the ring montage is used. This would also provide further information for comparing the ring montage with other tDCS arrays that are evaluated under similar experimental conditions. The final goal is to find a tDCS strategy that facilitates the improvement of motor performance by enhancing the excitability of stroke patient's motor pathways, improving this way the rehabilitation of his/her gait. This strategy will be combined in the future with a BCI system for motor neurorehabilitation of gait.

## 2. Materials and methods

### 2.1. Participants

Five volunteers between 20 and 30 years old participated in this study. None of them had any known neurological disease or any metallic implant. Despite these five participants comprise a small sample that cannot describe the statistical parameters from the whole population, results from this sample allow the separate analysis of each volunteer and the detection of the existence of any overall qualitative trend that is exhibited by all or most subjects.

### 2.2. Ethics statement

This work was carried out following the recommendations of the Office for Project Evaluations (Oficina Evaluadora de Proyectos: OEP) of Miguel Hernández University of Elche (Spain), which approved the experimental protocol. All subjects gave written informed consent in accordance with the Declaration of Helsinki.

### 2.3. EEG acquisition

The Enobio 32 system was used to acquire EEG data at a sampling rate of 500 Hz from 32 channels of the international 10/10 system: P7, P4, Cz, Pz, P3, P8, O1, O2, C2, F8, C4, F4, FP2, Fz, C3, F3, FP1, C1, F7, Oz, PO4, FC6, FC2, AF4, CP6, CP2, CP1, CP5, FC1, FC5, AF3, and PO3. In terms of software, the Neuroelectrics Instrument Controller (NIC) was used to obtain EEG data, while a MATLAB (MATLAB, RRID:SCR_001622) platform was used to record, process and analyze the EEG signals. In this case, the use of a higher density electrode array respect to the 10/20 system provides a more precise analysis through the signal processing of EEG data of higher spatial resolution. Nevertheless, the main purpose of obtaining data from more electrodes was to record brain activity for other possible future analyses, besides from the results that are presented in this work (Jurcak et al., 2007).

### 2.4. tDCS supply

**Figure 1:**
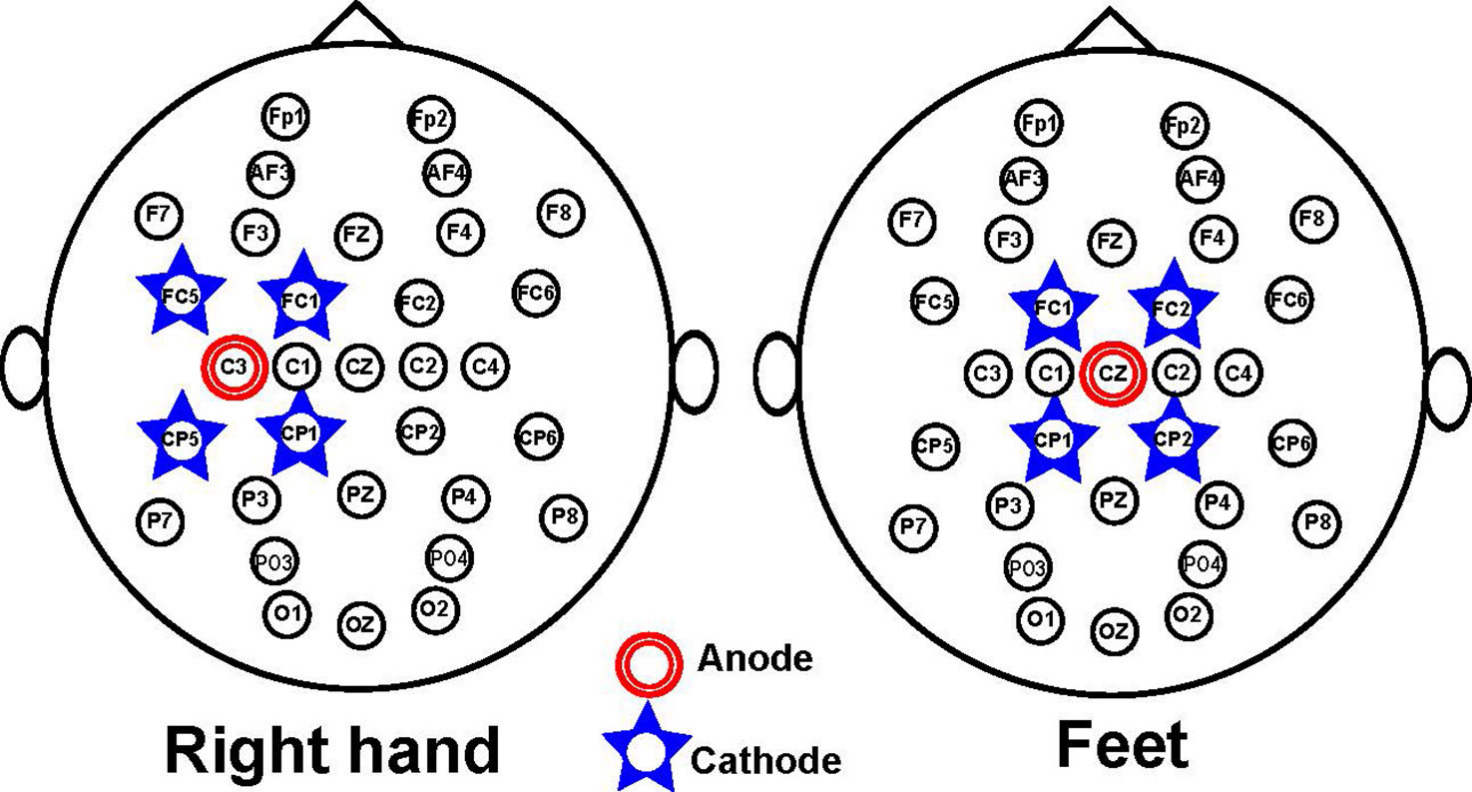
tDCS montage for stimulating the right-hand **(Left)** or feet **(Right)** motor cortex. The montage is shown in reference to the EEG electrode distribution.

The Starstim 8 system was used to provide anodal tDCS through electrodes with radius of 1 cm. In particular, five channels of the Starstim device were used to test two different tDCS montages of five electrodes: an electrode array that was aimed to stimulate the right-hand motor cortex, and another array that allowed the stimulation of the feet motor cortex. Both montages consisted of a central electrode (anode) over the target motor cortex and four return electrodes (cathodes) around it in order to provide relatively focused stimulation at the motor cortex. In case of the montage version that stimulated the right-hand motor cortex, the anode was located over C3, while the four return electrodes were placed on FC1, FC5, CP1, and CP5. In the montage version that stimulated the feet motor cortex, the anode and the four return electrodes were positioned on Cz, FC1, FC2, CP1, and CP2, respectively. These two possible arrays are presented in Figure 1. NIC software was used to trigger the stimulation.

### 2.5. Simulation

NIC software was used to produce the simulation of the electric field that was produced by the two tDCS arrays described in Section 2.4. The current values of the performed simulations represented the maximum current that was supplied in the experiments, i.e., 188 μA.

### 2.6. Experimental sessions

Each subject endured two blocks of four sessions, leaving at least 2 days between sessions in order to avoid tDCS cumulative effects. This intersession interval was selected based on the recommendation in Nitsche et al. (2008) of leaving 48 h to 1 week between stimulation sessions for tDCS protocols with long-lasting after-effects. In each block, only one of the following stimulation modalities was evaluated:

- **tDCS applied on the right-hand motor cortex**
  In this stimulation scheme, anodal tDCS was supplied for 10 min using the array described in Section 2.4 to stimulate the right-hand motor cortex. Ramps of 3 s were included at the beginning and the end of the stimulation pulse. Then, EEG was recorded while the subject followed instructions according to the presentation of visual cues: When a screen showed an arrow (5 s) pointing to the right or downward, the user had to imagine to move the right hand or feet, respectively, while a blank screen (4–4.5 s) indicated the user to remain at rest.
- **tDCS applied on the feet motor cortex**
  This modality had the same protocol for performing the experimental sessions than in the case of the previous tDCS scheme, with the only difference that tDCS was provided on the feet motor cortex instead of the right-hand motor area. For this purpose, the array described in Section 2.4 for stimulating the feet motor area was used.

**Figure 2:**
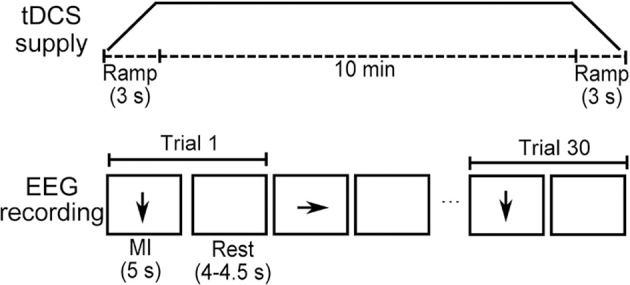
Temporal sequence of tDCS supply and one run of EEG recording.

**Figure 3:**
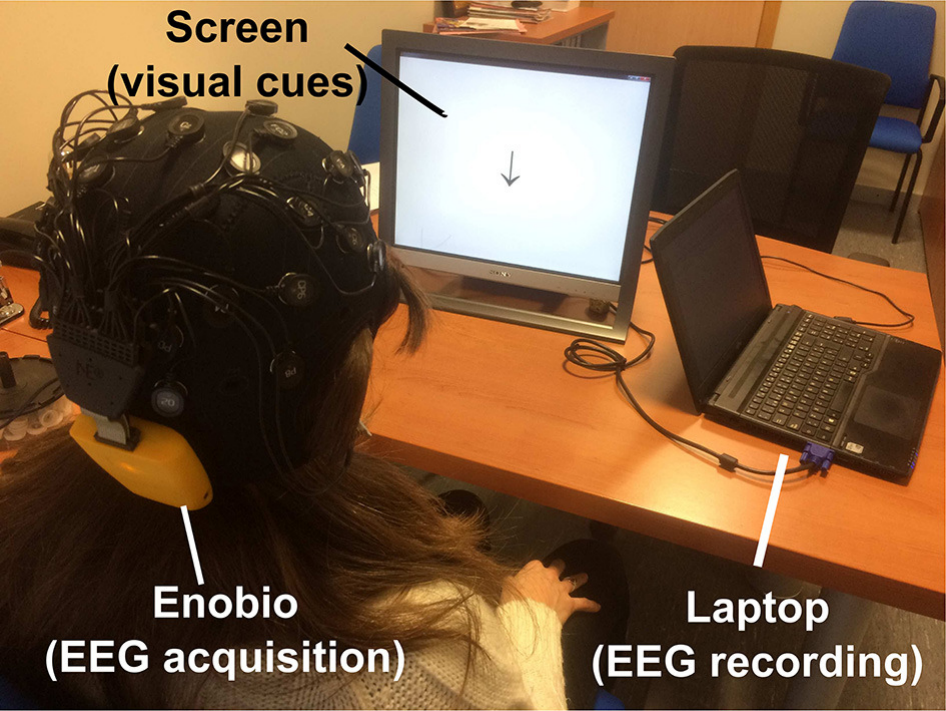
Experimental setup.

For both stimulation modalities, each session consisted of the supply of the 10-min tDCS followed by three runs of fifteen sequences of each kind of motor imagery with a corresponding rest period in each motor imagery trial. This means that in each run a total of fifteen trials (motor imagery plus rest) of right-hand motor imagery and fifteen trials of feet motor imagery were presented in random order. Figure 2 shows the temporal sequence of the tDCS supply and one run, while Figure 3 presents the experimental setup that was used while EEG was recorded. There were given breaks of approximately 3 min between runs.

In each of the four sessions from a block, a different current intensity of tDCS was provided with a possible value of 0 (sham stimulation), 63, 126, or 188 μA, which are denoted as D0, D1, D2, and D3, respectively. The order of the evaluation of the current intensities was counterbalanced between subjects, and the order of the presentation of the tDCS modalities was counterbalanced as well. The evaluated currents were selected as an approximation of the current strength that was required to produce current densities of 0 (D0), 0.02 (D1), 0.04 (D2), and 0.06 mA/cm^2^ (D3), which represent values within the range of the current densities that are used in most studies (0.029–0.08 mA/cm^2^) (Nitsche et al., 2008). In this case, the current density was estimated as the ratio of the current intensity *I* and the electrode area *A* (Nitsche et al., 2008), where *A* = π cm^2^. Note that Miranda et al. (2009) reported that small electrodes seem to be less efficient than larger ones and, thus, the traditional calculation *I*/*A* of current density is inaccurate. On the other hand, the effect of the supply of a constant *I*/*A* ratio with different size of electrodes has already been tested. In Nitsche et al. (2007), stimulating with an intensity of 0.1 mA and electrodes of 3.5 cm^2^ showed no significant difference on the elicited effects that were caused by the supply of 1 mA with 35 cm^2^ electrodes (an approximate current density of 0.029 mA/cm^2^). Hence, the lowest current density that was estimated and evaluated in the present study was 0.02 mA/cm^2^ in order to have a reference close to 0.029 mA/cm^2^, which is also the recommended maximum limit of stimulation in terms of comfort (Nitsche et al., 2003), despite the possible higher stimulus tolerability (Villamar et al., 2013).

### 2.7. EEG analysis

Once EEG was recorded, the accuracy was calculated on each session as the percentage of correct classifications of a specific kind of motor imagery (MI) and its rest condition with the objective of measuring how detectable the performance of MI was respect to the rest condition. Therefore, the accuracy indicated if the motor activity was enhanced on the session. In addition, the change of event-related synchronization (ERS), which refers to the level of the synchronization of neural activity that is associated to a performed task, was evaluated for the μ and β bands as the change of EEG power that was obtained during MI respect to the rest condition with the purpose of describing the EEG activity at each session. Then, comparison of the accuracy and ERS between sessions of a same modality were carried out through statistical analysis in order to find statistical differences in these metrics that were associated to the supply of different intensities of tDCS.

In order to obtain the accuracy and EEG band power in each session, the EEG signals went first through a preprocessing phase that relied on frequency filtering and artifact rejection to assure that EEG clean signals were used in further analysis. The initial part of this phase consisted in filtering EEG signals with a fourth-order bandpass Butterworth with cut-off frequencies of 5 and 45 Hz. Then, each trial was decomposed in 32 (i.e., equal to the number of channels) component signals through the independent component analysis (ICA) routine from EEGLAB toolbox (EEGLAB, RRID:SCR_007292), so blinking components could be visually detected. When an ICA component was contaminated with an artifact, it was filtered with an adaptive Wiener filter to estimate the artifact component in the ICA fragment, which also included brain activity information. Next, the estimated artifact was subtracted from the original ICA component and EEG signals were reconstructed. This preprocessing methodology allowed artifact removal with minimal EEG distortion (Heute and Guzmán, 2014). Reconstructed signals were inspected visually and, if the trial was still noisy, then it was discarded from the analysis.

After EEG was preprocessed, the signals of every trial were processed with a spatial filter that subtracted from Cz, C3, and C4 the mean of their four adjacent electrodes. In case of C3, the neighboring electrodes were FC1, FC5, CP1, and CP5. In contrast, for C4 the contiguous electrodes were FC2, FC6, CP2, and CP6. For Cz, the considered electrodes were FC1, FC2, CP1, and CP2. It should be mentioned that the brain activity from C3, Cz, and C4 was selected for the entire analysis because these channels are located in the regions of the motor cortex that may present major changes in relation to the application of tDCS. For example, C3 and Cz are located over the right-hand and feet motor cortex, respectively, and there is also connectivity at some frequencies between C3 and C4 (Pfurtscheller, 2001; Hamedi et al., 2016). Furthermore, there can be found ERS changes over these channels during right-hand and feet motor imagery, as well as contiguous ERS/ERD regions (Yi et al., 2013; Hamedi et al., 2016).

The resulting filtered signals of the three channels were divided into a MI epoch and its corresponding rest epoch, and they were separated depending on the kind of motor imagery that was performed during the trial. Hence, a set of MI and rest epochs are attained for the conditions of right-hand and feet MI. Next, the spectra after the second 2 was calculated for each epoch of MI and rest. Then, the following process was performed independently for the set of spectra of right-hand and feet MI to calculate accuracy and ERS.

#### 2.7.1. Accuracy

Fisher criterion (*F*) of the spectra on C3, C4, and Cz for MI and rest states was calculated as Saa and Gutierrez (2010):

(1) $$\begin{array}{r} F_{f}=\frac{{\left(\mu_{1,f}-\mu_{2,f}\right)}^{2}}{\delta_{1,f}^{2}+\delta_{2,f}^{2}}, \end{array}$$

where μ_1,*f*_ and μ_2,*f*_ correspond to the mean power at frequency *f* for conditions 1 (MI) and 2 (rest), respectively. On the other hand, δ_1,*f*_ is the standard deviation of power at frequency *f* for condition 1, while δ_2,*f*_ is the analog parameter for condition 2. Then, there were found the values of *f* where the maxima of *F* for C3, Cz, and C4 were located. Hence, a characteristic frequency was obtained for each of these channels in every session. Each characteristic feature represented the frequency in which MI and rest states were more separable at a particular channel, considering that the frequency where the maxima of *F* is found represents the frequency at which the mean values of MI and rest conditions have a greater difference and lower intra-condition variance.

Once characteristic features were obtained, 100 iterations were performed in which 30 random epochs were selected from the total epochs of both MI and rest conditions and, then, they were labeled in either MI or rest with help of a linear discriminant analysis (LDA) classifier. Such classifier was trained with the spectral power of C3, C4, and Cz from the remaining epochs (about 60) of the session at the characteristic frequency of the channels. After classification was conducted, the percentage of correct classifications from the 30 epochs was calculated in each iteration, so an accuracy distribution of 100 samples was obtained for each session.

It must be noted that the methodology for calculating accuracy relies on obtaining subject-specific features for classification in each session. This approach was used due to the known high inter-subject variability of brain activity, along with the expected high intra-subject variability of the sensorimotor rhythm modulation (Blankertz et al., 2010; Lightbody et al., 2014; Palaniappan et al., 2015), since no feedback about the MI performance was provided in order to avoid learning effects through the different sessions.

#### 2.7.2. ERS

ERS on C3, Cz, and C4 was obtained for each trial as the difference of the natural logarithms of mean spectral power of MI and rest states at μ or β bands:

(2) $$\begin{array}{rcl} ERS_{\mu } & = & \ln\overset{f=12}{\underset{9}{\sum }}\frac{S_{1}\left(f\right)}{4}-\ln\overset{f=12}{\underset{9}{\sum }}\frac{S_{2}\left(f\right)}{4} \end{array}$$

(3) $$\begin{array}{rcl} ERS_{\beta } & = & \ln\overset{f=30}{\underset{13}{\sum }}\frac{S_{1}\left(f\right)}{18}-\ln\overset{f=30}{\underset{13}{\sum }}\frac{S_{2}\left(f\right)}{18}, \end{array}$$

where *S*_1_(*f*) and *S*_2_(*f*) denote the spectral power of conditions 1 and 2, respectively, at frequency *f* within the μ or β frequency range at a specific channel. Note that in this case the logarithm is used with the objective of reducing the skewness of the spectral distribution.

After accuracy and ERS values were obtained for all sessions, statistical comparisons were made between the distributions that were obtained with the different current densities. Based on Pfurtscheller (2001), possible outliers were discarded from ERS measurements by removing the data that was out of the 95% confidence interval of the session. Also, in case of band power analysis, mean ERS of the sham session was subtracted from all sessions of the same stimulation modality in order to set the sham session as a reference with zero value. Statistical comparisons for accuracy relied on performing analysis of variance (ANOVA) (*p* < 0.001) with the aim of testing for significant differences on the accuracy between sessions of the same tDCS modality for each subject. If the test was significant, then multiple comparisons (*p* < 0.001) were made with Tukey-Kramer's method to identify which specific session was different from another. In case of ERS, *t*-tests (*p* < 0.05) were performed to compare the ERS between the sham session and other sessions in which tDCS was applied. Note that the significance value was lower in the accuracy statistical tests in order to take into account that the use of tDCS has a high cost. In contrast, the use of a higher significance threshold for ERS tests facilitated the observation of behavioral trends in ERS after the supply of tDCS. Also, a qualitative comparison was made with the outcomes from a previous study (Angulo-Sherman et al., 2017), which evaluated the effect of the stimulation with a tDCS montage that was aimed to influence the cortico-cerebellar motor path using the same current intensities from the present study.

## 3. Results

### 3.1. Distribution of the tDCS electric field based on simulations

**Figure 4:**
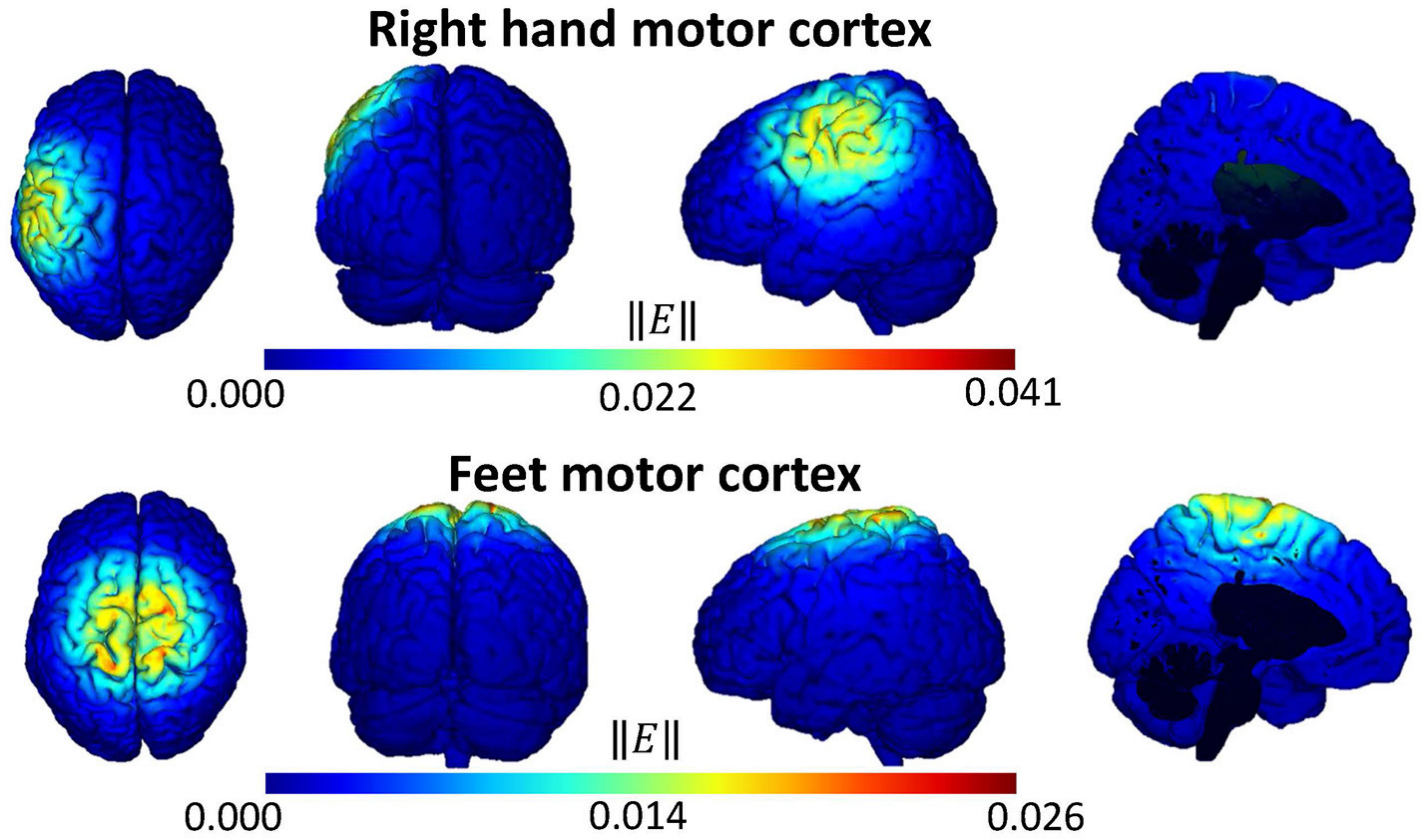
Simulation of the norm of the electric field ||*E*|| (V/m) that is generated by tDCS supply on the right-hand **(Top)** and feet **(Bottom)** motor cortex.

Figure 4 presents, from left to right, the superior, posterior and right side views of the brain, as well as an image from the left hemisphere. These views show the norm of the electric field that is produced while stimulating with an intensity of 188 μA for both tDCS modalities. In general, the top and bottom images reflect that the cortical areas that were the target of the stimulation are affected by a focused electric field. In case of targeting the right-hand motor cortex (top), it can be seen that the stimulation is focused over C3 and its nearby areas. Thus, there is relatively focused stimulation of the right-hand motor cortex. Note that the configuration of the stimulation with this montage version involves four return electrodes, including FC1 and CP1. These two electrodes are used in the spatial filtering of C3 and Cz, so possible effects could be expected at both electrodes. On the other hand, the focus of the electric field can be found over Cz and its surrounding regions when the tDCS is targeting the feet motor cortex (bottom), which reflects the stimulation of this region. The configuration of this montage version includes four return electrodes: FC1, CP1, FC2, and CP2, which are used in the spatial filtering process of C3, Cz, and C4. Hence, the effect in this area can affect the EEG activity observed at the three electrodes, though not necessarily in a significant manner.

### 3.2. Accuracy changes related to tDCS supply

**Figure 5:**
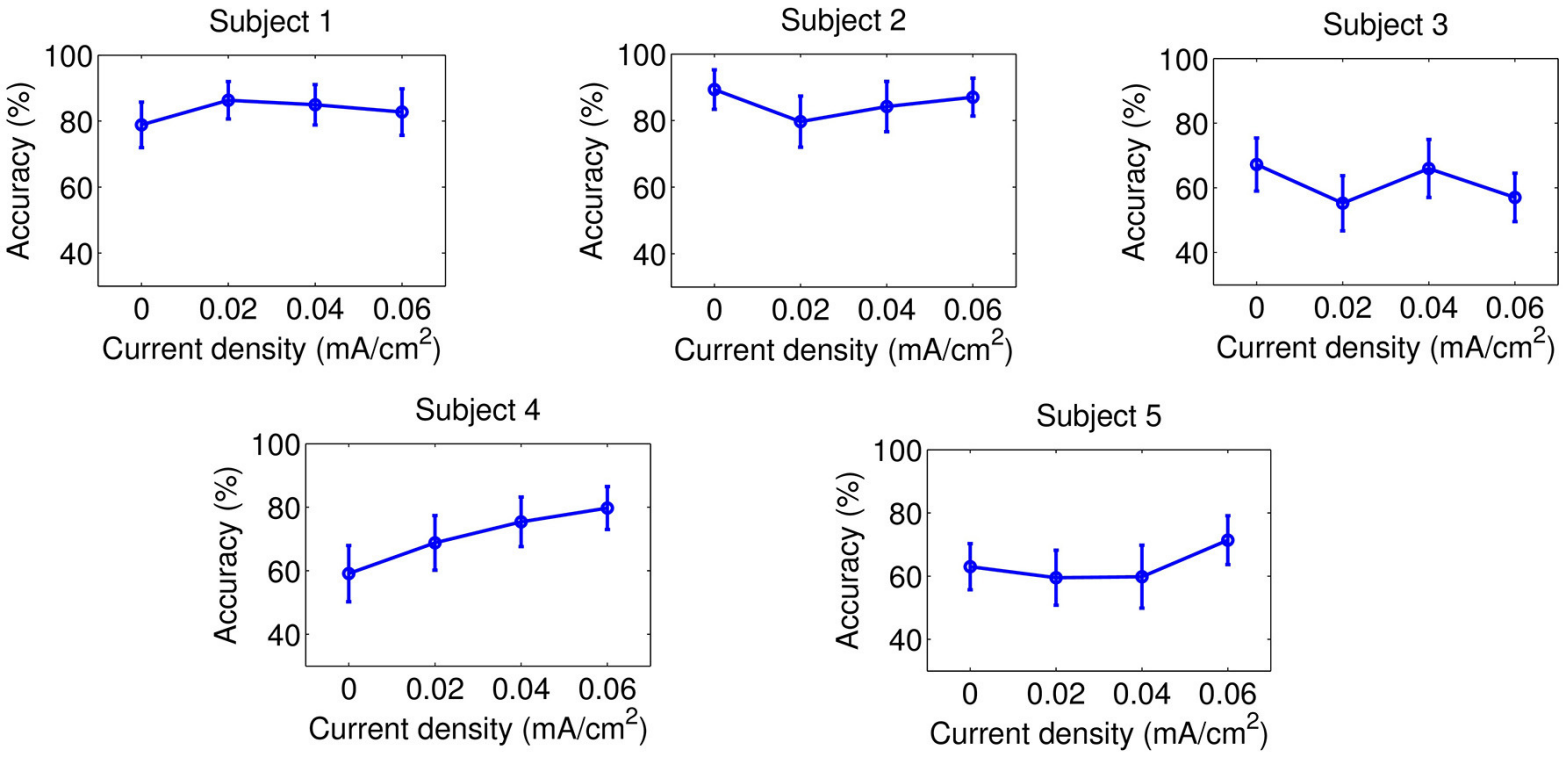
Accuracy of right-hand MI after the stimulation of the right-hand motor cortex. Each point represents the mean accuracy for a particular session, while the error bars show the standard deviation.

**Figure 6:**
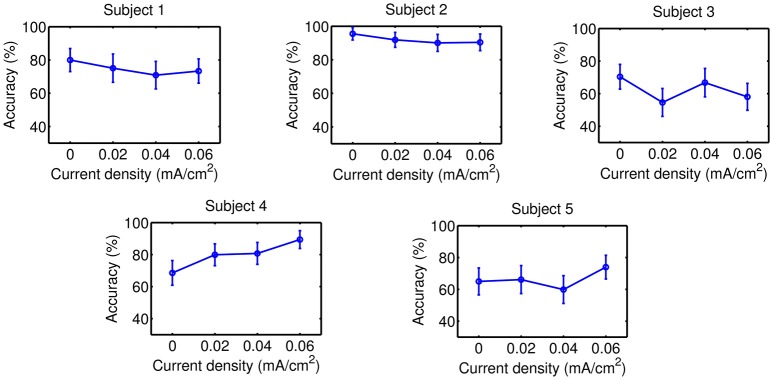
Accuracy of feet MI after the stimulation of the right-hand motor cortex. Each point represents the mean accuracy for a particular session, while the error bars show the standard deviation.

**Figure 7:**
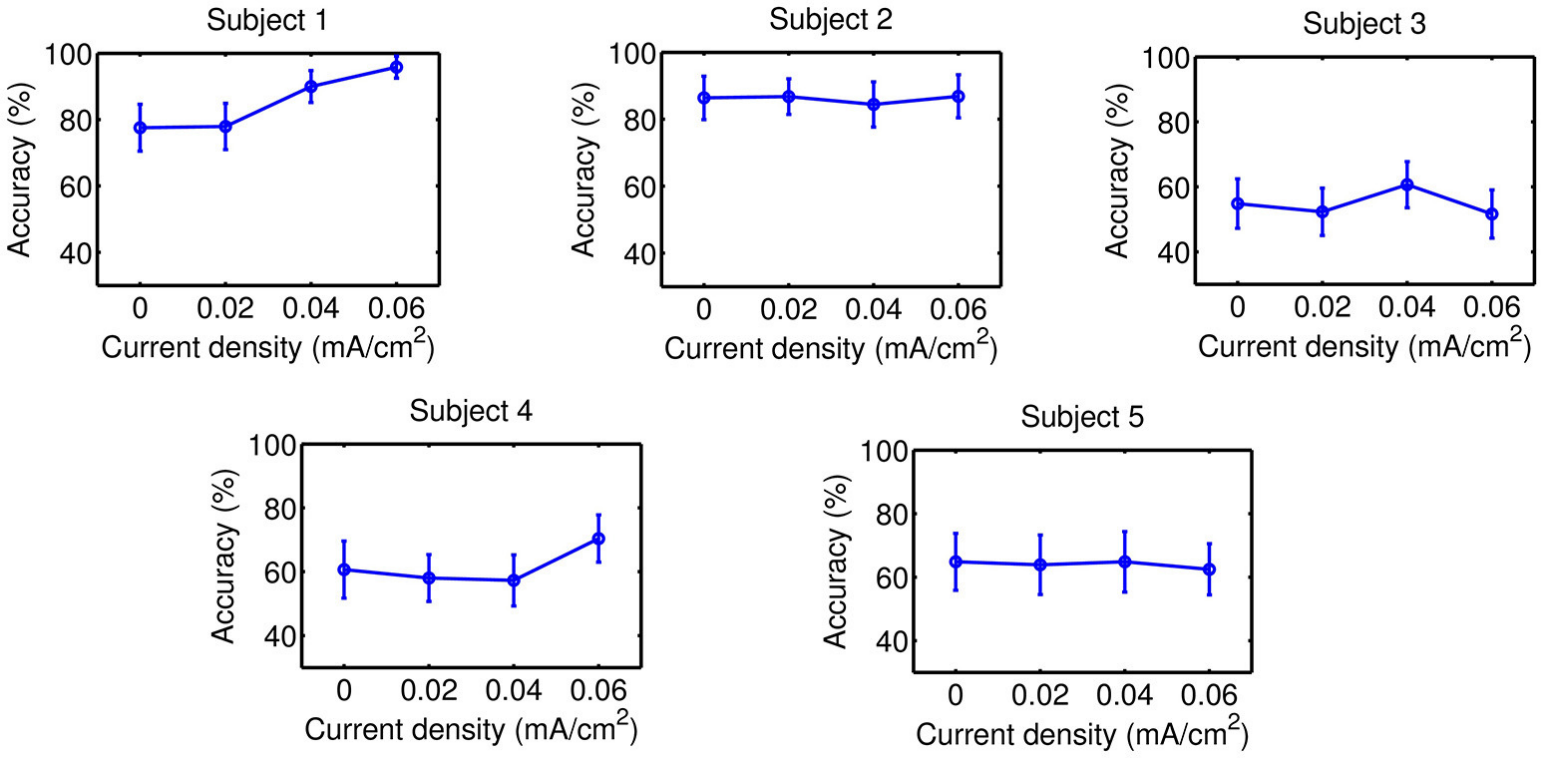
Accuracy of right-hand MI after the stimulation of the feet motor cortex. Each point represents the mean accuracy for a particular session, while the error bars show the standard deviation.

**Figure 8:**
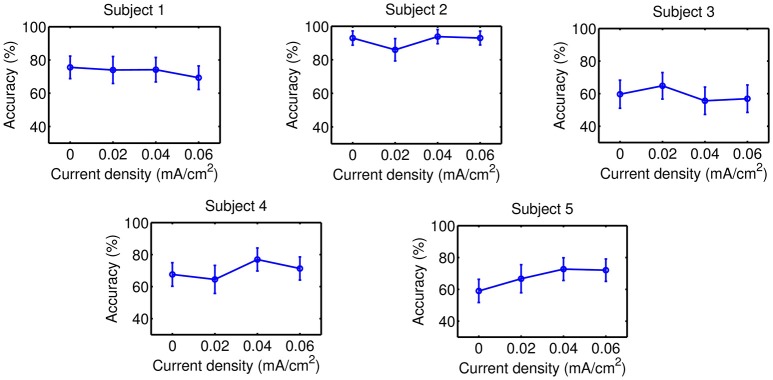
Accuracy of feet MI after the stimulation of the feet motor cortex. Each point represents the mean accuracy for a particular session, while the error bars show the standard deviation.

Accuracy results are presented on Figures 5–**8** for each kind of MI, subject and stimulation modality, while statistical analysis are included in Tables 1, 2. In the tables, first and second columns indicate the MI and number of subject that are analyzed, respectively. The third and fourth columns show the results of the statistical analysis, which consist of the *p*-value from the ANOVA tests and the comparisons between the different current densities that were found significant. For example, in the Table 1 and for the right-hand MI of Subject 1, it was observed that the sham stimulation (D0) provided a significant lower accuracy than the current densities of 0.02 (D1) and 0.04 (D2), and 0.06 (D3) mA/cm^2^, while D3 showed also a significant lower accuracy compared to D1. This is denoted as D0<D1, D2, D3; D3<D1. Global trends in the results of accuracy are described next for each tDCS modality.

**Table 1:** Statistical tests of accuracy when tDCS is applied over the right-hand motor cortex.

**Motor imagery**	**S**	**ANOVA (*p*-value)**	**Multiple comparisons (*p* < 0.001)**
Right hand	1	3.99 × 10^−15^	D0<D1, D2, D3; D3<D1
	2	2.02 × 10^−21^	D1<D2<D0; D1<D3
	3	3.50 × 10^−29^	D1, D3<D0, D2
	4	8.81 × 10^−57^	D0<D1<D2<D3
	5	7.25 × 10^−24^	D0, D1, D2<D3
Feet	1	1.76 × 10^−14^	D1, D2, D3<D0; D2<D1
	2	3.42 × 10^−17^	D1, D2, D3<D0
	3	6.12 × 10^−40^	D1, D3<D0, D2
	4	9.42 × 10^−68^	D0<D1, D2<D3
	5	9.43 × 10^−27^	D2<D0, D1<D3

**Table 2:** Statistical tests of accuracy when tDCS is applied over the feet motor cortex.

**Motor imagery**	**S**	**ANOVA (*p*-value)**	**Multiple comparisons (*p* < 0.001)**
Right hand	1	1.78 × 10^−90^	D0, D1<D2<D3
	2	0.0191	–
	3	5.30 × 10^−18^	D0, D1, D3<D2
	4	4.91 × 10^−31^	D0, D1, D2<D3
	5	0.2051	–
Feet	1	1.88 × 10^−8^	D3<D0, D1, D2
	2	7.30 × 10^−30^	D1<D0, D2, D3
	3	5.45 × 10^−14^	D0, D2, D3<D1
	4	7.73 × 10^−27^	D0<D2; D1<D3<D2
	5	3.48 × 10^−36^	D0<D1<D2, D3

#### 3.2.1. tDCS applied on the right-hand motor cortex

Figures 5, 6, in addition to Table 1, present the results of this stimulation scheme. In case of right-hand MI, D3 was the current intensity that seemed to provide improvements for more subjects (three out of five users: S1, S4, and S5) compared to sham stimulation. However, this same current appeared to worsen the classification for S3 respect to when no stimulation was applied. Then, there is no beneficial trend for any of the applied currents, considering that none of the evaluated current values increased accuracy for most subjects without worsening the classification for a subject. Likewise, no current intensity was observed to have an incremental trend on accuracy for feet MI.

#### 3.2.2. tDCS applied on the feet motor cortex

Results of this tDCS modality are shown on Figures 7, 8, as well as on Table 2. For right-hand MI, it can be seen that three out of five users improved their accuracy with either D2 or D3 respect to D0, so an optimal current within D2 or D3 might be considered to possibly improve accuracy. Nevertheless, the variability in the possible optimal current intensity does not reveal any clear incremental trend in accuracy. In case of feet MI, none of the evaluated current values was associated to a significant increase of accuracy for most subjects compared to sham stimulation.

Based on the accuracy results that were previously described, it seems that no favorable trends in the detection of right-hand or feet MI were obtained with any of the evaluated current intensities, either when the tDCS was applied over the right-hand motor cortex or when it was supplied over the feet motor cortex.

In order to provide an insight about the obtained results in context with another montage, a comparison with the results obtained with the montage in Angulo-Sherman et al. (2017) is made. In that work, the effect of applying the same current intensities as in this study, but with a montage that was aimed to stimulate the cortico-cerebellar motor path, over the classification of right-hand and feet MI was evaluated. That study suggested that placing the anode over the cortical motor area and the anode over the left cerebellum may enhance motor imagery detection. In particular, D3 seemed to show potential of improving the classification of right-hand MI when the anode was used to target the right-hand motor area, considering that four out of five volunteers improved about 10% their accuracy for right-hand MI, while the remaining subject showed no significant effects due to the stimulation. However, that study was still exploratory and results were not conclusive yet. It should be acknowledged that in case of the 4 × 1 ring montage, the expected optimal currents would be probably higher, considering that the inter-electrode distance is smaller, which would lead to higher current dispersion in the external regions of the head and lower current density at increasing depth (Faria et al., 2011). Thus, a fairer comparison between montages would include montage-specific currents in order to provide more similar current densities at a specific point of the motor cortex. However, this comparison still provides information about the effects that are found at low tDCS currents for both montages. It should be noted that, despite of the small sample of five subjects that was used on both studies, the results provide information about the possible existence of the improvement or worsening accuracy trends after the stimulation at a particular current strength respect to the case when sham tDCS is supplied.

### 3.3. ERS changes related to tDCS supply

**Figure 9:**
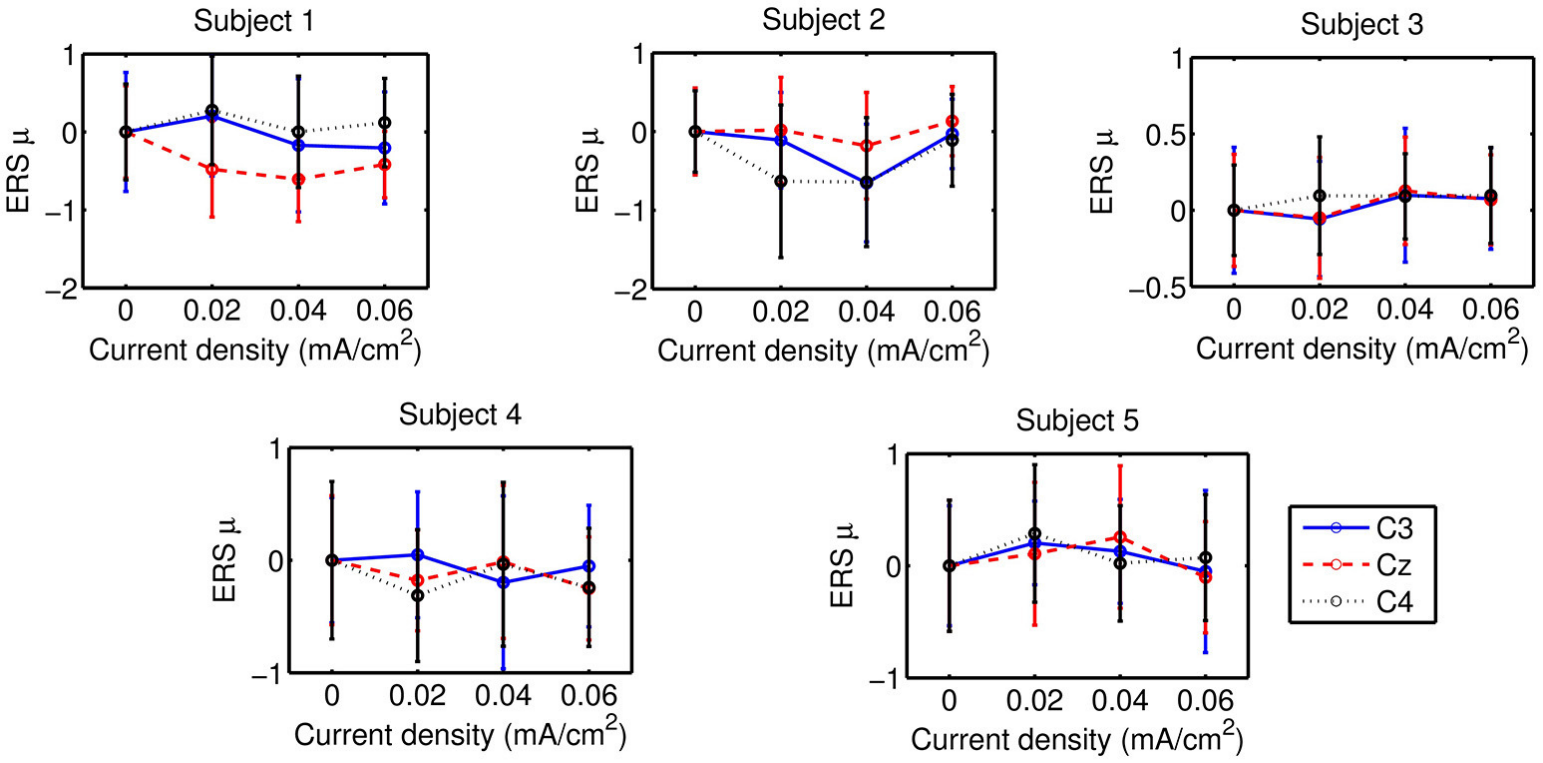
ERS associated to right-hand MI in the μ-band after supplying tDCS over the right-hand motor cortex. Each point represents the mean ERS for a particular session, while the error bars show the standard deviation.

Statistical results of ERS are presented in Tables 3, 4 for the tDCS modalities that target the motor cortex of the right-hand and the feet, respectively. In these tables, the first column indicates the number of subject whose results are tested, while the second column shows the channel that is analyzed. The third column includes the significant results from the *t*-tests that compare ERS of right-hand MI from the sham stimulation against other sessions where different intensities of tDCS were applied. The column is divided in two subcolumns that provide the results of the analysis for μ and β bands, using the same notation that was used for reporting the results of accuracy. Similarly, the fourth column presents the results of the ERS *t*-tests for feet MI. It should be mentioned that ERS data have a high variance that suggests ERS changes between sessions are subtle. For example, Figure 9 shows the mean μ-band power in C3, Cz, and C4 that is associated in each session to the performance of right-hand MI after tDCS is supplied over the right-hand motor cortex. There it can be seen that the standard deviation is high. Hence, these results only serve as a guide to describe subtle variations and no abrupt ERS changes should be inferred. General trends of ERS when a current is applied respect to the sham session are described next.

**Table 3:** ERS statistical results for the case when tDCS is applied over the right-hand motor cortex.

**S**	**Channel**	**Right hand**		**Feet**	
		**μ band**	**β band**	**μ band**	**β band**
1	C3	–	–	D1, D2, D3>D0	–
	Cz	D1, D2, D3<D0	D1, D2, D3<D0	D2>D0	D3>D0
	C4	D1>D0	–	D2, D3>D0	D2, D3>D0
2	C3	D2<D0	D1>D0; D2<D0	D1, D2, D3<D0	D2, D3<D0
	Cz	–	D1, D2<D0	D2<D0	D1, D2, D3<D0
	C4	D1, D2<D0	D1, D2<D0	D1, D2<D0	D1, D2<D0
3	C3	–	D2>D0	D1, D2, D3>D0	–
	Cz	D2>D0	–	D1, D3>D0	–
	C4	D2, D3>D0	–	–	D2>D0
4	C3	–	D2, D3<D0	D1<D0	D1, D2, D3>D0
	Cz	D1, D3<D0	–	–	D2, D3<D0
	C4	D1, D3<D0	D3<D0	–	D1<D0
5	C3	D1>D0	D1, D2, D3>D0	D1, D3>D0	D1, D2>D0
	Cz	D2>D0	D3>D0	–	D3<D0
	C4	D1>D0	D1, D3>D0	–	D1<D0

**Table 4:** ERS statistical results for the case when tDCS is applied over the feet motor cortex.

**S**	**Channel**	**Right hand**		**Feet**	
		**μ band**	**β Xband**	**μ band**	**β band**
1	C3	D2, D3<D0	D1, D2, D3<D0	–	–
	Cz	D1, D3>D0	D1>D0	D1, D3>D0	–
	C4	D1>D0; D2, D3<D0	D3<D0	D2<D0	D3>D0
2	C3	D1, D2>D0	D1, D2>D0; D3<D0	D1, D2>D0	D1, D2>D0
	Cz	D2>D0	D2>D0	D1, D2>D0	D1>D0; D3<D0
	C4	D2>D0	D1, D3<D0; D2>D0	D2>D0	D1, D3<D0; D2>D0
3	C3	–	–	D2>D0; D3<D0	D2, D3<D0
	Cz	D3<D0	D3<D0	D3<D0	–
	C4	–	–	D3<D0	–
4	C3	–	–	–	–
	Cz	D1, D2<D0	D1<D0	D1>D0	D1>D0; D2<D0
	C4	D1>D0	D3<D0	–	D1<D0; D2>D0
5	C3	D1, D2>D0	D3>D0	D1<D0	D2<D0
	Cz	D2>D0	D1, D2<D0	D2>D0	D1>D0
	C4	D1>D0	–	D1, D3<D0	–

When the significant differences that indicate if D0 has either a higher or lower ERS respect to other currents are observed in the tables, it can be observed that the results for the different MI conditions vary among the subjects, so no clear trends can be distinguished for all subjects at specific channels. Despite of the focused stimulation, no attenuation trend in the ERS was found directly on C3 when the stimulation was applied over the right-hand motor cortex, or on Cz when the tDCS target was the feet motor cortex. On the other hand, most cases that present a significant difference between the ERS from the sham session and other currents follow the same trend. For example, in Table 3 it can be seen that the Subject 1 presents lower ERS at Cz when either D1, D2, and D3 are applied compared to D0. Also, it should be noted that some users can present in general the same incremental or decremental trends in Cz, C3, and C4, such as the Subjects 3 and 4 for the case of right-hand MI in Table 3, which suggests the presence of ERS differences that are spatially nonspecific.

## 4. Discussion

In general terms, results from the accuracy analysis showed that the supply of tDCS with a 4 × 1 ring montage over either the right-hand or feet motor cortex showed no global improvement trends on the detection of right-hand or feet motor imagery for the evaluated current range (up to 188 μA). Note that in Nitsche et al. (2007) a current as low as 100 μA was reported to induce significant after-effects with another cephalic montage. Moreover, results from the 4 × 1 ring montage were compared against the results with the same current range from this study, but with a montage that was aimed to affect the motor cortex and the cerebellum in order to influence the motor cortico-cerebellar path (Angulo-Sherman et al., 2017). The stimulation with that montage seemed to show potential of enhancing motor imagery detection in a small sample on a exploratory study, but results were still not conclusive. In the case of the ring montage, no improvements on motor imagery have been observed, which could be related to the expected higher dispersion of the electric field in the more external regions of the head under the electrodes (Faria et al., 2011). Then, the evaluated current range (estimated through the *I*/*A* ratio) is probably of a lower intensity for small electrodes than the one required to provide the desired current density values (Miranda et al., 2009), since no trend is observed on the elicited effects of tDCS. This would support the idea that higher currents are needed to evaluate the ring montage, as it is performed in Roy et al. (2014) and reviewed in Villamar et al. (2013). The lack of observed effects in the results of this study appears to indicate the possible superiority of other montages with less focalization at lower current intensities, such as in Nitsche et al. (2007) and Angulo-Sherman et al. (2017), but also that a fairer comparison should be performed with the appropriate parameters for each tDCS electrode array. Note that not only the current intensity is relevant for obtaining optimal results. For example, some studies report the delay of the tDCS effects when using HD-tDCS arrays (Kuo et al., 2013; Baxter et al., 2014), which shows that the optimal time for evaluating a task after the supply of stimulation could be relevant for the evaluation of each tDCS montage. Hence, a more extensive independent evaluation of tDCS electrode configurations should be performed for improving the comparison of tDCS montages. It should be mentioned that, despite of the small sample size from this study, it was possible to detect the high variability of accuracy changes within subjects, which shows that the stimulation protocol provides no general increment on the accuracy of classification of motor imagery. Hence, it is not necessary to perform any more experiments on a larger sample, but future research with the ring montage should use higher current intensities in order to possibly improve accuracy results and, once the optimal current range is identified, use the best current parameters to compare this montage against other tDCS electrode configurations in a fair manner.

ERS analysis showed that the spectral EEG changes due to the tDCS supply had a high variance, so the detected ERS differences between the various current intensities were associated to subtle EEG synchronization modulations. No trends were observed in the ERS of the three channels, despite the expected effect over the electrodes that are localized over the targeted motor cortex. In addition, there were cases where some subjects showed either the increase or decrease of ERS on the three analyzed channels (Cz, C3 of C4) for the same tDCS modality. This probably reflects that the detected significant ERS differences are related to some underlying activity that is not spatially specific, despite the focused stimulation shown on the simulation of the electric field that is induced by the montage. These results are consistent with the lack of observed effects of the stimulation over classification accuracy.

In summary, the supply of currents up to approximately 0.2 mA provided no significant improvements in the detection of either right-hand or feet MI. However, this study is useful and necessary for the comparison among montages at very low intensities, considering that other montages seem to present some significant effects at those values. Anyway, further evaluation at higher intensities and the estimation of more accurate current densities at different regions of the motor cortex would be required to allow comparison of electrode arrays for enhancing motor activity. This is necessary for improving the stimulation strategies before their possible implementation in future motor neurorehabilitation systems.

## 5. Conclusion

The accuracy of the classification of feet and right-hand motor imagery was evaluated in a small sample of subjects after applying different values of current (up to 188 μA) with a 4 × 1 ring montage. Results show that no improvement trends of classification were found. Considering that a montage of two electrodes and greater inter-electrode distance, such as in Nitsche et al. (2007) or Angulo-Sherman et al. (2017), seems to have a significant effect at a current value of similar magnitude, it appears that the current intensity required to elicit significant effects was underestimated for the 4 × 1 montage by the use of the ratio of the current intensity and the electrode size to estimate the expected current density. Therefore, it appears that there is higher dispersion of the electric field over the outer regions of the head for this montage. Thus, a lower current density was applied over the motor cortex, which was the target of the stimulation. The lack of trends in ERS at particular electrodes despite the focused stimulation, along with the similarity of the significant ERS changes over different regions of the motor cortex in some cases, suggests that there were no spatially-specific EEG changes related to the administration of tDCS. This supports the conclusion that the current intensity was not high enough to elicit observable after-effects. The fact that the same current intensity provides different results when targeting the motor cortex with different montages, indicates that fairer comparisons between montages should be performed with montage-specific parameters. In the case of the 4 × 1 ring montage, higher currents should be evaluated in order to obtain significant after-effects. Nevertheless, the obtained results are valuable for making comparisons between tDCS montages at low current intensities. Despite the sample was small, the lack of any incremental accuracy trend for the subjects at a specific current intensity indicates that no more experiments should be performed with this exact same protocol for the ring montage, but higher current intensities must be evaluated.

## Author contributions

IA, MR, and EI designed the study. IA and MR collected the data and performed the simulations. IA analyzed data and interpreted the results. JA actively contributed as director of the work. All the authors participate conceiving the experiments and drafting the manuscript. All authors read and approved the final manuscript.

### Conflict of interest statement

The authors declare that the research was conducted in the absence of any commercial or financial relationships that could be construed as a potential conflict of interest.

## References

[B1] Ang K. K. Guan C. Phua K. S. Wang C. Teh I. Chen C. W. (2012). Transcranial direct current stimulation and EEG-based motor imagery BCI for upper limb stroke rehabilitation, in 2012 Annual International Conference of the IEEE Engineering in Medicine and Biology Society, eds Cauwenberghs G. Weiland J. D. (San Diego, CA: IEEE), 4128–4131. 10.1109/EMBC.2012.6346875 23366836

[B2] Angulo-Sherman I. N. Rodríguez-Ugarte M. Sciacca N. Iáñez E. Azorín J. M. (2017). Effect of tDCS stimulation of motor cortex and cerebellum on EEG classification of motor imagery and sensorimotor band power. J. Neuroeng. Rehabil. 14:31. 10.1186/s12984-017-0242-1 28420382 PMC5395900

[B3] Baxter B. S. Edelman B. Zhang X. Roy A. He B. (2014). Simultaneous high-definition transcranial direct current stimulation of the motor cortex and motor imagery, in 2014 36th Annual International Conference of the IEEE Engineering in Medicine and Biology Society, ed Ying L. (Chicago, IL: IEEE), 454–456. 10.1109/EMBC.2014.6943626 25569994

[B4] Bikson M. Datta A. Elwassif M. (2009). Establishing safety limits for transcranial direct current stimulation. Clin. Neurophysiol. 120:1033. 10.1016/j.clinph.2009.03.018 19394269 PMC2754807

[B5] Bikson M. Grossman P. Thomas C. Zannou A. L. Jiang J. Adnan T.. (2016). Safety of transcranial direct current stimulation: evidence based update 2016. Brain Stimul. 9, 641–661. 10.1016/j.brs.2016.06.004 27372845 PMC5007190

[B6] Blankertz B. Sannelli C. Halder S. Hammer E. M. Kübler A. Müller K.-R.. (2010). Neurophysiological predictor of SMR-based BCI performance. Neuroimage 51, 1303–1309. 10.1016/j.neuroimage.2010.03.022 20303409

[B7] Datta A. Truong D. Minhas P. Parra L. C. Bikson M. (2012). Inter-individual variation during transcranial direct current stimulation and normalization of dose using MRI-derived computational models. Front. Psychiatry 3:91. 10.3389/fpsyt.2012.00091 23097644 PMC3477710

[B8] Faria P. Hallett M. Miranda P. C. (2011). A finite element analysis of the effect of electrode area and inter-electrode distance on the spatial distribution of the current density in tDCS. J. Neural Eng. 8:066017. 10.1088/1741-2560/8/6/066017 22086257 PMC3411515

[B9] Foerster Á. Rocha S. Wiesiolek C. Chagas A. P. Machado G. Silva E.. (2013). Site-specific effects of mental practice combined with transcranial direct current stimulation on motor learning. Eur. J. Neurosci. 37, 786–794. 10.1111/ejn.12079 23279569

[B10] Hamedi M. Salleh S. Noor A. (2016). Electroencephalographic motor imagery brain connectivity analysis for BCI: a review. Neural Comput. 28, 999–1041. 10.1162/NECO_a_00838 27137671

[B11] Heute U. Guzmán A. S. (2014). Removing “cleaned” eye-blinking artifacts from EEG measurements, in Signal Processing and Integrated Networks (SPIN), 2014 International Conference on (Noida: IEEE), 576–580. 10.1109/SPIN.2014.6777020

[B12] Jurcak V. Tsuzuki D. Dan I. (2007). 10/20, 10/10, and 10/5 systems revisited: their validity as relative head-surface-based positioning systems. Neuroimage 34, 1600–1611. 10.1016/j.neuroimage.2006.09.024 17207640

[B13] Kuo H.-I. Bikson M. Datta A. Minhas P. Paulus W. Kuo M.-F.. (2013). Comparing cortical plasticity induced by conventional and high-definition 4 × 1 ring tDCS: a neurophysiological study. Brain Stimul. 6, 644–648. 10.1016/j.brs.2012.09.010 23149292

[B14] Lightbody G. Galway L. McCullagh P. (2014). The brain computer interface: Barriers to becoming pervasive, in Pervasive Health, eds Holzinger A. Ziefle M. Röcker C. (London: Springer), 101–129.

[B15] Minhas P. Bikson M. Woods A. J. Rosen A. R. Kessler S. K. (2012). Transcranial direct current stimulation in pediatric brain: a computational modeling study, in Engineering in Medicine and Biology Society (EMBC), 2012 Annual International Conference of the IEEE (San Diego, CA: IEEE), 859–862. 10.1109/EMBC.2012.6346067 PMC3641645 23366028

[B16] Miranda P. C. Faria P. Hallett M. (2009). What does the ratio of injected current to electrode area tell us about current density in the brain during tDCS? Clin. Neurophysiol. 120, 1183–1187. 10.1016/j.clinph.2009.03.023 19423386 PMC2758822

[B17] Neuper C. Scherer R. Reiner M. Pfurtscheller G. (2005). Imagery of motor actions: differential effects of kinesthetic and visual–motor mode of imagery in single-trial EEG. Cogn. Brain Res. 25, 668–677. 10.1016/j.cogbrainres.2005.08.014 16236487

[B18] Nitsche M. A. Cohen L. G. Wassermann E. M. Priori A. Lang N. Antal A.. (2008). Transcranial direct current stimulation: state of the art 2008. Brain Stimul. 1, 206–223. 10.1016/j.brs.2008.06.004 20633386

[B19] Nitsche M. A. Doemkes S. Karakoese T. Antal A. Liebetanz D. Lang N.. (2007). Shaping the effects of transcranial direct current stimulation of the human motor cortex. J. Neurophysiol. 97, 3109–3117. 10.1152/jn.01312.2006 17251360

[B20] Nitsche M. A. Liebetanz D. Lang N. Antal A. Tergau F. Paulus W. (2003). Safety criteria for transcranial direct current stimulation (tDCS) in humans. Clin. Neurophysiol. 114, 2220–2222. 10.1016/S1388-2457(03)00235-9 14580622

[B21] Palaniappan R. Andrews S. Sillitoe I. P. Shira T. Paramesran R. (2015). Improving the feature stability and classification performance of bimodal brain and heart biometrics, in SIRS, eds Thampi S. M. Bandyopadhyay S. Krishnan S. Li K.-C. Mosin S. Ma M. (Cham: Springer), 175–186.

[B22] Pfurtscheller G. (2001). Functional brain imaging based on ERD/ERS. Vision Res. 41, 1257–1260. 10.1016/S0042-6989(00)00235-2 11322970

[B23] Reis J. Fritsch B. (2011). Modulation of motor performance and motor learning by transcranial direct current stimulation. Curr. Opin. Neurol. 24, 590–596. 10.1097/WCO.0b013e32834c3db0 21968548

[B24] Roy A. Baxter B. He B. (2014). High-definition transcranial direct current stimulation induces both acute and persistent changes in broadband cortical synchronization: a simultaneous tDCS–EEG study. IEEE Trans. Biomed. Eng. 61, 1967–1978. 10.1109/TBME.2014.2311071 24956615 PMC4113724

[B25] Saa J. F. D. Gutierrez M. S. (2010). EEG signal classification using power spectral features and linear discriminant analysis: a brain computer interface application, in Eighth Latin American and Caribbean Conference for Engineering and Technology, eds Larrondo Petrie M. M. Bermudez M. Alvarez D. Esparragoza I. E. (Arequipa: LACCEI), 1–7.

[B26] Sharma N. Baron J.-C. (2013). Does motor imagery share neural networks with executed movement: a multivariate fMRI analysis. Front. Hum. Neurosci. 7:564. 10.3389/fnhum.2013.00564 24062666 PMC3771114

[B27] Villamar M. F. Volz M. S. Bikson M. Datta A. DaSilva A. F. Fregni F. (2013). Technique and considerations in the use of 4 × 1 ring high-definition transcranial direct current stimulation (HD-tDCS). J. Visual. Exp. 10.3791/50309 PMC3735368 23893039

[B28] Woods A. J. Martin D. M. (2016). Clinical research and methodological aspects for tDCS research, in Transcranial Direct Current Stimulation in Neuropsychiatric Disorders, eds Brunoni A. Nitsche M. Loo C. (Cham: Springer), 393–404. 10.1007/978-3-319-33967-2_26

[B29] Yi W. Qiu S. Qi H. Zhang L. Wan B. Ming D. (2013). EEG feature comparison and classification of simple and compound limb motor imagery. J. Neuroeng. Rehabil. 10:106. 10.1186/1743-0003-10-106 24119261 PMC3853015

